# Pro-Apoptotic Effect of Zeolitic Imidazolate Framework-8 (ZIF-8)-Loaded Dihydromyricetin on HepG2 Cells

**DOI:** 10.3390/molecules27175484

**Published:** 2022-08-26

**Authors:** Xiao Mi, Juan Lu, Mingran Dong, Yang Lou, Xia Zhan, Xi Chen

**Affiliations:** 1 Institute of Medicinal Plant Development, Chinese Academy of Medical Sciences, Peking Union Medical College, Beijing 100193, China; 2 Key Laboratory of Cleaner Production and Integrated Resource Utilization of China National Light Industry, Beijing Technology and Business University, Beijing 100048, China

**Keywords:** zeolitic imidazolate framework, ZIF-8, dihydromyricetin, HepG2 cells, apoptosis

## Abstract

Dihydromyricetin (DHM) has garnered attention due to its promising antitumor activity, but its low bioavailability restricts its clinical application. Thus, developing nano-drug delivery systems could enhance its antitumor activity. We prepared DHM@ZIF-8 nanoparticles using the zeolite imidazole framework-8 (ZIF-8) as a carrier loaded with dihydromyricetin. A series of characterizations were performed, including morphology, particle size, zeta potential, X-single crystal diffraction, ultraviolet spectroscopy, infrared spectroscopy, and Brunauer–Emmett–Teller (BET). The in vitro release characteristics of DHM@ZIF-8 under pH = 5.0 and pH = 7.4 were studied using membrane dialysis. The antitumor activity and pro-apoptotic mechanism of DHM@ZIF-8 were investigated through CCK-8 assay, reactive oxygen species (ROS), Annexin V/PI double-staining, transmission electron microscopy, and Western blot. The results depicted that DHM@ZIF-8 possessed a regular morphology with a particle size of 211.07 ± 9.65 nm (PDI: 0.19 ± 0.06) and a Zeta potential of −28.77 ± 0.67 mV. The 24 h drug releasing rate in PBS solution at pH = 7.4 was 32.08% and at pH = 5.0 was 85.52% in a simulated tumor micro acid environment. DHM@ZIF-8 could significantly enhance the killing effect on HepG2 cells compared to the prodrug. It can effectively remove ROS from the tumor cells, promote apoptosis, and significantly affect the expression of apoptosis-related proteins within tumor cells.

## 1. Introduction

Liver cancer is one of the most common global malignancies, with a five-year survival rate of 15–17% [1]. Statistical data reveal that liver cancer ranks in the top five most prevalent cancers worldwide and it’s also the third leading cause of cancer-related deaths [2]. Currently, the main clinical treatments involve surgical resection, liver transplantation, and systemic chemotherapy, but the prognosis for patients remains extremely poor. The chemotherapy drugs target tumor cells and severely affect normal cells [3]. Therefore, targeted drugs addressing the tumor cells remain a shared goal of drug research.

Dihydromyricetin (DHM) is a compound causing damage to tumor cells without any significant toxicity to normal cells [4,5]. It is abundant in *Ampelopsis grossedentata* (Hand.-Mazz.) W.T. Wang (Vitaceae) (20–30%, *w*/*w*), whose tender stems and leaves are widely used as vine tea in south China [6,7]. It has exhibited several pharmacological activities, including anti-inflammatory [8], hypoglycemic [9], antiviral [10], antimicrobial [11], and anti-allergic effects [12]. Besides, DHM suppresses hepatocellular carcinoma [13,14], non-small cell lung carcinoma [15], ovarian cancer [16], and melanoma [8]. DHM treatment inhibits cell proliferation, induces apoptosis and autophagy, and regulates redox balance in liver cancer cells; thus, exhibiting remarkable anticancer effects [7,17]. However, poor water solubility and extremely low oral bioavailability of DHM have considerably restricted its clinical application [18].

It is vital to select a suitable nanocarrier to improve the bioavailability of DHM. Metal organic frameworks (MOFs) are a new class of hybrid materials with metal ions and organic ligands bridged with large specific surface area, tunable pore size, and biodegradability, offering efficient drug loading [19,20]. Zeolite imidazole framework-8 (ZIF-8) is an emerging nano-metal organic framework (NMOF) with a simple synthesis strategy, easy functionalization, high loading capacity, and pH-responsive degradation. Nanoparticles of ZIF-8 have been widely used for DNA, protein, and drug delivery, and ZIF-8-based nano-delivery systems can avoid premature drug release and offer the possibility of enhancing drug bioavailability [21].

Therefore, we prepared a DHM drug delivery system using ZIF-8 as the carrier. The results revealed that this system could be better released within a micro-acidic environment, effectively enhancing the pro-apoptotic effect of DHM on HepG2 cells and increasing the expression of apoptosis-related proteins.

## 2. Results and Discussion

### 2.1. Synthesis and Characterization of DHM@ZIF-8

**Figure 1:**
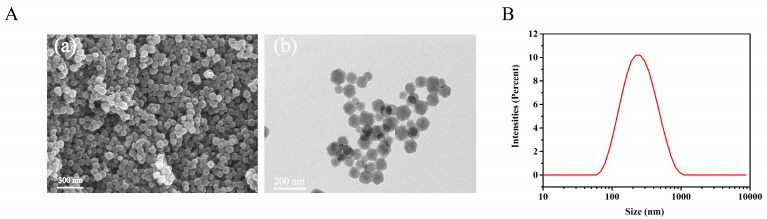
(**A**) SEM image (**a**) and TEM image (**b**) of DHM@ZIF-8. (**B**) Particle size distribution of DHM@ZIF-8.

**Figure 2:**
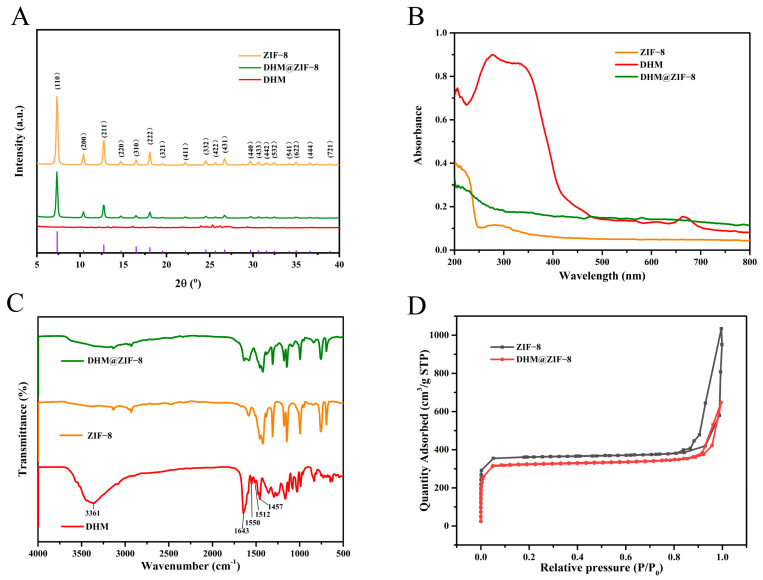
(**A**) XRD maps of ZIF-8, DHM and DHM@ZIF-8. (**B**) UV absorption profiles of ZIF-8, DHM, and DHM@ZIF-8. (**C**) IR spectra of ZIF-8, DHM, and DHM@ZIF-8. (**D**) N_2_ adsorption-desorption isotherms of ZIF-8 and DHM@ZIF-8.

As shown in Figure 1A, the SEM (a) and TEM (b) images depicted that DHM@ZIF-8 possessed a relatively uniform particle size. A Malvern particle sizer determined the hydrated particle size of DHM@ZIF-8 (Figure 1B) as 211.07 ± 9.65 nm (PDI: 0.19 ± 0.06), and the Zeta potential was measured as −28.77 ± 0.67 mV. The elemental dispersive spectrum (EDS) showed that the content of oxygen(O) element in DHM@ZIF-8 increased significantly compared with ZIF-8 due to DHM loading (Figure S1). The crystalline structures of ZIF-8 and DHM@ZIF-8 were examined through XRD (Figure 2A), and the crystalline structure of the prepared DHM@ZIF-8 was the same as that of ZIF-8. Therefore, the loading of DHM did not destroy the crystal structural integrity of ZIF-8. The UV-Vis detection results (Figure 2B) indicate that no absorption band of DHM was present within the spectrum of DHM@ZIF-8, indicating that DHM was loaded in ZIF-8. In addition, the loading process of the DHM nanoplatform was determined using Fourier transform infrared (FT-IR) spectroscopy (Figure 2C). Comparing the spectra of DHM and DHM@ZIF-8, it was observed that the characteristic peaks at 3361 cm^−1^ (OH) and those of benzene rings at 1643 cm^−1^, 1550 cm^−1^, 1512 cm^−1^, and 1457 cm^−1^ disappeared within the spectrum of DHM. These results indicated that the characteristic peaks of DHM were covered due to the physical shielding of DHM with ZIF-8, which further suggests that the DHM was loaded inside ZIF-8. In addition, we used Brunauer–Emmett–Teller (BET) to determine the specific surface area of DHM@ZIF-8 nanostructures (Figure 2D), and ZIF-8 was utilized as the control under similar conditions. The BET-specific surface area of ZIF-8 was 1232.59 m^2^/g, and the BET-specific surface area was 980.98 m^2^/g. The decrease in the specific surface area also indicated the successful loading of the drug.

### 2.2. Drug Loading Rate and Drug Releasing Rate

**Figure 3:**
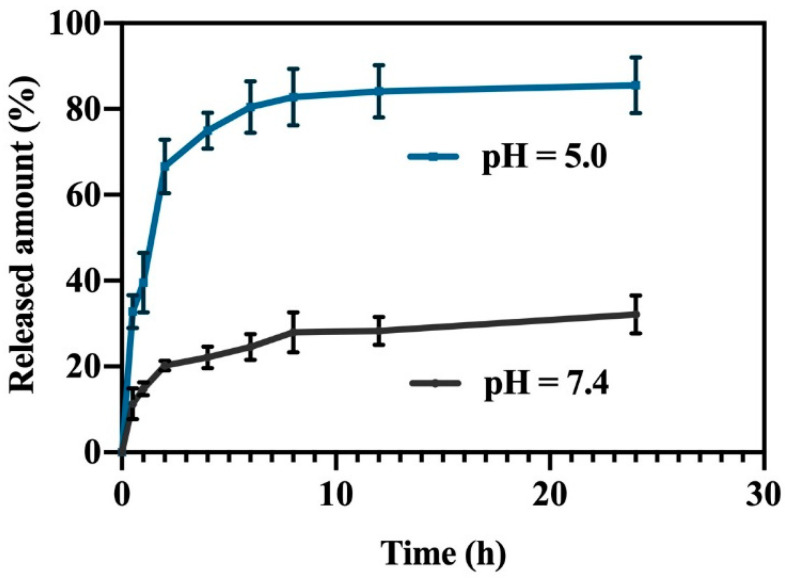
Release curves of DHM in PBS buffered solution at pH = 5.0 or 7.4. Data are representative of three independent experiments.

As shown in Figure S2, to assess the loading and release behavior of ZIF-8 on DHM, we established a standard curve of DHM solution at 290 nm. We investigated the drug loading under different ratios of drug and nanoparticles, and found that the ratio of 1:1 can significantly increase the drug loading compared with the ratio of 1:3 drug to carrier. However, when using the 3:1 ratio of drug to carrier for feeding, the increase of drug loading was not significant, so we choose the 1:1 ratio for follow-up experiments (Figure S3). We finally measured that the loading rate of DHM@ZIF-8 was 19.17 ± 1.24%. Then, we measured the drug release behavior under different pH values (pH = 5.0 or pH = 7.4). As described in Figure 3, the 24 h drug releasing rate was 32.08% in a PBS solution simulating the pH of the normal physiological environment. In contrast, the final drug releasing rate was 85.52% within a PBS solution simulating the pH of the slightly acidic environment of the tumor. This exhibited that ZIF-8 had good acid-responsive release properties and was a good platform for tumor drug delivery.

### 2.3. Cytotoxicity Assay

**Figure 4:**
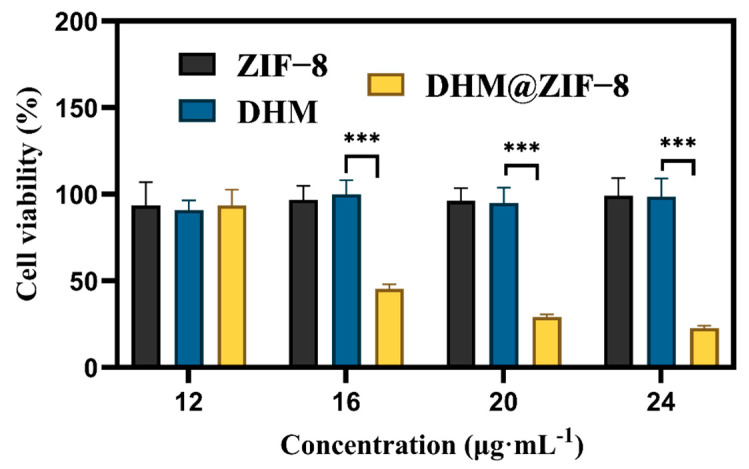
Cell viability of HepG2 cells treated with different concentrations of samples for 12 h. Data are representative of four independent experiments. *** *p* < 0.001 vs. DHM.

The CCK-8 assay was used to determine the cell survival rate at different doses to investigate the lethal effect of the DHM@ZIF-8 drug delivery system on HepG2 (Figure 4). It was observed that ZIF-8 and DHM failed to kill HepG2. In contrast, a significant decrease in cell survival was observed when HepG2 cells were treated using a nano-delivery system consisting of ZIF-8 loaded DHM, demonstrating the superior killing effect of the DHM@ZIF-8 drug delivery system over HepG2 tumor cells.

### 2.4. Live/Dead Cell Staining

**Figure 5:**
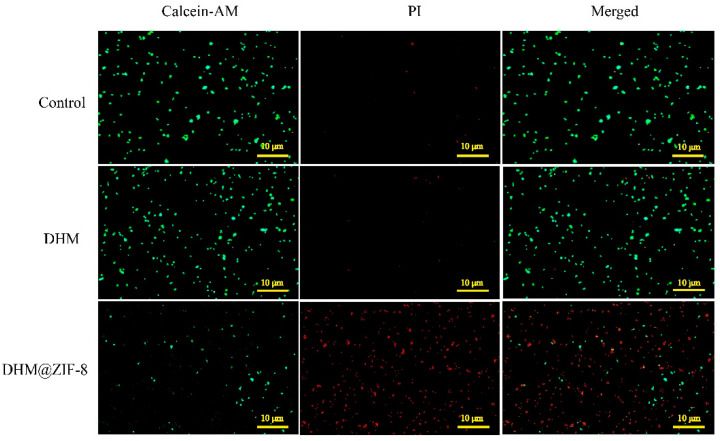
Fluorescence microscopy images were used after processing with DHM or DHM@ZIF-8, and all the images were scaled to 10 µm. Living cells were represented by green fluorescence and dead cells were represented by red fluorescence.

Calcein AM enters the cell and is hydrolyzed by endogenous esterases in living cells to produce calcein, a polar molecule with a strong negative charge not permeating the cell membrane. It is retained in the cell to emit strong green fluorescence. Therefore, only live cells are stained with solid green fluorescence; dead cells are not stained or stained very weakly. The nucleic acid red fluorescent dye propidium iodide (PI) can only stain the dead cells where the integrity of the cell membrane is disrupted. Therefore, both are used to detect cell activity and cytotoxicity. As shown in the Figure 5, after the action of DHM on HepG2 cells by the live/dead cell staining assay, no significant killing effect on tumor cells was observed compared to the control group. When ZIF-8 was used as a nanoplatform for delivering DHM, it caused a massive tumor cell death, as evidenced by a significant enhancement of red fluorescence in the field of view. This demonstrated the excellent antitumor effect of the DHM@ZIF-8 nanodelivery system.

### 2.5. Determination of ROS Content

**Figure 6:**
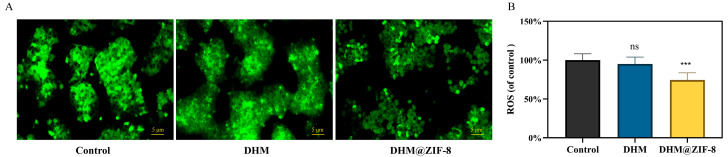
(**A**) ROS fluorescence microscopy images after being processed with DHM or DHM@ZIF-8. The scaling bars for all images are 5 μm. (**B**) ROS content of the DHM or DHM@ZIF-8 group was determined using a fluorescent enzyme marker. Data are representative of six independent experiments. ns represents no statistical significance, *** *p* < 0.001 vs. control.

Redox imbalance induced by ROS excess or deficiency is a predisposing factor in disease pathogenesis, including tumorigenesis and progression. Cancer cells require a higher level of ROS than normal cells. In case the level of ROS is lower than the minimum requirement for the cellular response, cancer cells cannot grow naturally [22,23]. DHM has previously disrupted the redox balance in HepG2 cells through ROS, inhibiting their proliferation and inducing apoptosis [7,24]. We measured the intracellular reactive oxygen species (ROS) levels in HepG2 cells treated with DHM and DHM@ZIF-8. As shown in Figure 6A,B, our results indicated that HepG2 cells had highly expressed ROS and that DHM could not scavenge ROS well at lower doses, while the intracellular delivery of DHM using ZIF-8 as a vector significantly affects the production of ROS in tumor cells, thus affecting tumor cell growth.

### 2.6. Cell Apoptosis

**Figure 7:**
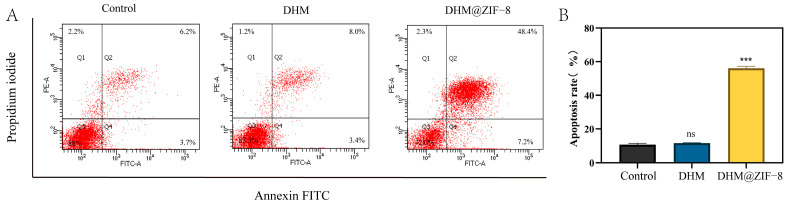
(**A**) Apoptosis after treatment with DHM or DHM@ZIF-8. (**B**) Apoptosis rate. Data are representative of three independent experiments. ns represents no statistical significance, *** *p* < 0.001 vs. control.

The facilitation of apoptosis in tumor cells is one of the mechanisms by which many natural product drugs exert their antitumor effects [25]. To demonstrate that DHM@ZIF-8 enhances the apoptosis-inducing effect of DHM on tumor cells, we measured the apoptosis rates of different drugs using flow cytometry. As shown in the Figure 7A,B, the apoptosis-inducing effect of DHM on tumor cells was not evident at lower doses, with an apoptosis rate of only 11.6 ± 0.26%. In contrast, the apoptosis rate of tumor cells was substantially increased when DHM@ZIF-8 was used for the same duration of action, with an apoptosis rate of 56.07 ± 1.27%. The effective delivery of ZIF-8 resulted in a substantial increase in the effect of DHM in promoting apoptosis.

### 2.7. Apoptotic Cell Morphology Observed Using the Transmission Electron Microscope

**Figure 8:**
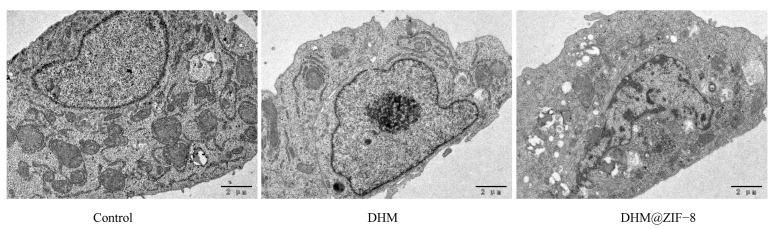
Cell morphology after treatment with DHM or DHM@ZIF-8. Scale bars = 2 μm.

To visualize the changes inside the cells more closely, we used transmission electron microscopy to observe the morphology of the cells under different treatment conditions. As shown in the Figure 8, the cell morphology of the blank group was normal, and the nuclear chromatin was aggregated after treatment of HepG2 cells with DHM, suggesting that DHM treatment had a certain degree of effect on the tumor cells, yet it was not apparent. In contrast, the HepG2 cells treated with DHM@ZIF-8 had irregular nuclear morphology, severe cytoplasmic bubbling, and severe apoptosis of tumor cells.

### 2.8. Western Blot

**Figure 9:**
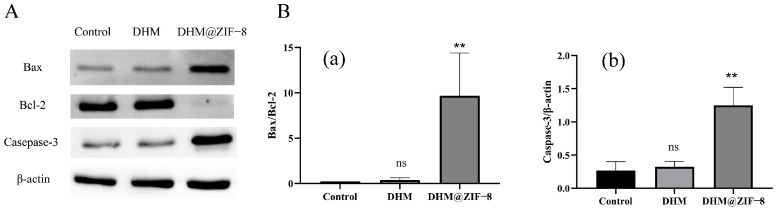
(**A**) Detection of apoptosis-associated proteins in cells treated with DHM or DHM@ZIF-8. (**B**) Quantitative analysis of protein expression. (**a**) The Bax/Bcl-2 ratio and (**b**) The expression of Caspase-3. Data are representative of three independent experiments. ns represents no statistical significance, ** *p* < 0.01 vs. control.

Bax and Bcl-2 are two mutually antagonistic apoptosis regulatory proteins and members of the Bcl-2 family. The ratio of Bax/Bcl-2 determines whether a cell survives or apoptoses [26,27]. Under normal conditions, Bax and Bcl-2 remain in the proper ratio, and when apoptosis occurs, the Bax/Bcl-2 ratio is altered. As the expression of Bax increases, mitochondrial membrane permeability improves and activates Caspase family proteins, such as Caspase-3. Previous studies have shown that DHM induced apoptosis in HepG2 cells, increased Bax/Bcl-2 ratio and casepase-3 protein expression [4]. We used Western blot to detect the expression of the anti-apoptotic protein Bcl-2 and the pro-apoptotic proteins Bax and Caspase-3 to ascertain the facilitating effect of DHM@ZIF-8 on apoptosis in HepG2 cells at the protein level (Figure 9A). We measured the Bax/Bcl-2 ratio and the expression of Caspase-3 (Figure 9B). The results depicted that DHM@ZIF-8 significantly elevated the Bax/Bcl-2 ratio and the expression of Caspase-3 compared to the original DHM, revealing that DHM@ZIF-8 significantly enhanced the pro-apoptotic ability of DHM.

## 3. Materials and Methods

### 3.1. Materials and Reagents

Zinc nitrate hexahydrate (Zn(NO_3_)_2_·6H_2_O, 99%) was obtained from Tianjin FuChen Chemical Reagent Co., Ltd. (Tianjin, China). 2-methylimidazole (C_4_H_6_N_2_, 98%) was purchased from TCI Development Co., Ltd. (Shanghai, China). Dihydromyricetin was secured from Shanghai Winherb Medical Technology Co., Ltd. (Shanghai, China) with purity >99%; PAN Seratech GmbH, Aidenbach, Germany, provided fetal bovine serum. Cell Counting Kit-8 (CCK-8) and Calcein/PI Cell Viability/Cytotoxicity Assay Kit were supplied by Shanghai Beyotime Biotechnology Co., Ltd. (Shanghai, China). Annexin V-FITC Apoptosis Detection Kit and 2′,7′-Dichlorodihydrofluorescein diacetate was purchased from Beijing Solarbio Science & Technology Co., Ltd. (Beijing, China). The antibodies were purchased from Abcam (Shanghai) trading Co., Ltd. (Shanghai, China). Absorbance and fluorescence intensity were detected using the TECAN spark multifunctional microplate reader. Fluorescence images were taken with a Nikon Tclipse Ts2R fluorescence inverted microscope. The apoptosis assay was performed with the BD FACSaira II flow cytometer. The apoptotic morphology was photographed using a JEM-1200EX (JEOL, Tokyo, Japan) transmission electron microscope.

### 3.2. Cell Lines

HepG2 cells were provided by the Cell Resource Centre, Institute of Basic Medical Sciences, Chinese Academy of Medical Sciences and were cultured in 5% CO_2_ at 37 °C.

### 3.3. Synthesis of DHM@ZIF-8

#### 3.3.1. Synthesis of ZIF-8

2.975 g of zinc nitrate hexahydrate and 3.284 g of 2-methylimidazole (1:4 molar ratio) were weighed. Zinc nitrate hexahydrate was placed in a 1000 mL round bottom flask, and 2-methylimidazole was put in a 500 mL conical flask. 200 mL of methanol was added separately and stirred at 1000 r/min for 5 min. The 2-methylimidazole solution was poured into the stirring zinc nitrate hexahydrate solution, which was stirred for 1.5 h, left for 24 h, and the precipitate was collected through centrifugation at 8000 r/min and washed with fresh methanol. The resultant precipitate was kept in a vacuum drying oven and dried overnight at 60 °C to acquire ZIF-8.

#### 3.3.2. Synthesis of DHM@ZIF-8

DHM was loaded with a post-adsorption method. Different proportions of dihydromyricetin and ZIF-8 nanoparticles were weighed and dispersed within 50 mL of methanol, stirred at 600 r/min for 24 h, washed several times using methanol, centrifuged, and the precipitate was obtained and dried overnight in a vacuum drying oven.

### 3.4. Characterization of DHM@ZIF-8

SEM images were obtained using a ZEISS Sigma 500 scanning electron microscope. TEM micrographs were collected from an FEI Talos F200s 200 kV, a field emission transmission electron microscope. The elemental dispersive spectra (EDS) were obtained by Japanese electron field emission scanning electron microscope JSM-7800F.The particle size was determined by Malvern Mastersizer 2000 laser particle sizer. XRD was recorded on a D8 ADVANCE X-ray polycrystalline diffractometer. The UV–vis absorption spectra were evaluated through a UV-3600i Plus spectrophotometer. FT-IR was secured on a Thermo Scientific Nicolet iS5N with KBr pellets. Nitrogen adsorption/desorption analyses were performed with a Micromeritics ASAP 2460.

### 3.5. Determination of Drug-Loading Rate and Drug-Releasing Rate

The loading and releasing rates were measured using the Agilent Technologies Cary series UV-vis spectrophotometer at 290 nm absorption. A standard curve for DHM was developed. A specific mass of nanoparticles was weighed in a volumetric flask, and a small amount of 0.9% hydrochloric acid-methanol solution was utilized to disrupt the structure of ZIF-8 to release the drug. The absorbance was measured after determining the volume. The drug loading rate was calculated with the following equation.

$$\mathrm{DLE}\;(\%)=\frac{W1}{\mathrm{Wt}}\;\times \;100\%$$

W1 is the weight of the drug loaded within the nanoparticles, and Wt is the total weight of the nanoparticles.

The drug releasing rate was measured with dynamic membrane dialysis. A mass of DHM@ZIF-8 was weighed, dispersed using phosphate buffered solution (PBS) of different pH, and kept in a dialysis bag (MWCO: 3500 Da). Then, the dialysis bags were placed in centrifuge tubes containing 25 mL of different pH (pH = 5.0 or pH = 7.4) on a shaker at 37 °C and centrifuged at 100 r/min. Subsequently, 0.5 mL of PBS was removed at a set time point and replenished using the same volume of fresh PBS. The absorbance was measured with a UV spectrophotometer, and the cumulative release rate of the drug was determined.

### 3.6. Role of In Vitro Anti-Hepatocellular Carcinoma Cells HepG2

#### 3.6.1. Cytotoxicity Assay

HepG2 cells were inoculated within 96-well plates at a density of approximately 8 × 10^3^ cells per well and then incubated at 37 °C for 24 h. The culture medium was removed, and then the cells were incubated in a cell culture medium with ZIF-8, DHM and DHM@ZIF-8 (the concentrations of ZIF-8 and DHM were determined using the DHM@ZIF-8 content, having a final concentration of 12–24 µg/mL of DHM) for 12 h. At the end of the incubation, the medium was aspirated and discarded, 100 µL of the CCK-8 dilution solution was added, and the incubation was continued for 1 h. At the end of the incubation, the absorbance was measured at 450 nm with a standard enzyme.

#### 3.6.2. Living/Dead Cell Staining

HepG2 cells were inoculated at 2.5 × 10^4^ cells per well density in 24-well culture dishes and incubated for 24 h. After cell apposition, the culture medium containing DHM or DHM@ZIF-8 (containing 20 µg/mL of DHM) was replaced. In the blank group, the fresh culture medium without the drug was replaced with three samples within each group. After interacting with the drug, the residual drug was washed off using PBS, and 0.5 mL Calcein-AM/PI solution was added to stain the cells for 30 min. The cell fluorescence images were obtained using an inverted fluorescent microscope.

### 3.7. Determination of ROS Content

HepG2 cells were inoculated at 8 × 10^3^ cells per well in 96-well black culture plates having transparent bottoms and incubated for 48 h. After cell apposition, the medium was replaced using a culture medium containing DHM or DHM@ZIF-8 (containing 20 µg/mL of DHM). The blank group was replaced with a fresh cell culture medium without a drug. After 12 h of drug interaction, the cells were stained with 2,7-dichlorofluorescein diacetate (DCFH-DA) solution used to measure intracellular ROS production for 10 min, gently washed, photographed using a fluorescence microscope, and quantified using a fluorescent enzyme marker.

### 3.8. Cell Apoptosis

HepG2 cells were inoculated within cell culture flasks at a density of 4 × 10^5^ and incubated against the wall for 48 h. The medium was replaced with a cell medium containing DHM or DHM@ZIF-8 (containing 20 µg/mL of DHM). In the blank group, the medium was replaced using a fresh cell medium without drugs, and three samples were repeated in parallel within each group. Cells were stained after 12 h of incubation based on the kit instructions, and then the samples were assayed with flow cytometry.

### 3.9. Observation Using the Transmission Electron Microscope

A bottle of cells grown to approximately 90% fusion was passaged at a 1:4 ratio and well-cultured for 24 h. The cell medium containing DHM or DHM@ZIF-8 (containing 20 µg/mL of DHM) was used, and for the blank group, a fresh cell medium without the drugs was used. After incubation for 12 h the cells were fixed with 2.5% glutaraldehyde at 4 °C for 30 min. Then, the cells were gently scraped off using a cell scraper, packed in 1.5 mL EP tubes, and centrifuged at 3000 r/min to collect the cell pellets. After enrichment of sufficient cell clusters, 1 mL fresh glutaraldehyde fixative was added at 4 °C overnight. The samples were photographed after pre-treatment with the machine.

### 3.10. Apoptosis-Related Proteins Detected Using Western Blot

HepG2 cells were treated with a medium having DHM or DHM@ZIF-8 (containing 20 µg/mL of DHM) for 12 h and gently rinsed using PBS. The appropriate volume of RIPA lysate was added (protease inhibitor was added minutes before use). Moreover, total protein was extracted and processed based on the manufacturer’s instructions. Proteins were resolved with SDS–PAGE and transferred onto the PVDF membranes (Millipore, Tullagreen Carrigtwohill, County Cork, Ireland). After being blocked with 5% nonfat dry milk, the membranes were incubated with primary antibodies at 1:1000, followed by three 10 min washes, and incubated with the HRP-second antibody at a dilution of 1:1000. The imaging was obtained through chemiluminescence and analyzed using the “Image-pro plus 6.0 (Media Cybernetics, MD, USA)”.

### 3.11. Statistics

Data are expressed as means ± SD. Statistical evaluation was performed by one-way ANOVA. ns represents no statistical significance, ** is *p* < 0.01 and *** is *p* < 0.001.

## 4. Conclusions

In this work, a simple method was used to synthesize a DHM nano-drug delivery system that significantly enhances the killing effect of the prodrug DHM on HepG2. DHM@ZIF-8 has a smaller particle size and characterized by a narrow particle size distribution. Using various characterization tools, the drug DHM was successfully loaded into ZIF-8. Due to its acid-responsive properties, ZIF-8 could selectively release DHM in response to the micro-acidic environment of the tumor, thereby significantly boosting the killing effect on HepG2 cells. It was further observed that the delivery of ZIF-8 also enhanced the ability of DHM to scavenge ROS from tumor cells, facilitating an elevation in the apoptotic rate and changes in apoptotic morphology. This resulted in an increase in the Bax/Bcl-2 ratio and high production of the pro-apoptotic protein caspase-3 within tumor cells. As a result, DHM@ZIF-8 provides a possible strategy for more efficient treatment of cancer.

## Data Availability

The data presented in this study are available in supplementary material.

## Supplementary Materials

The following supporting information can be downloaded at: https://www.mdpi.com/article/10.3390/molecules27175484/s1, Figure S1: The elemental dispersive spectrum (EDS) of ZIF-8 and DHM@ZIF-8; Figure S2: Standard curve of DHM solution detected by UV spectrophotometer at 290 nm; Figure S3: The loading efficiency of DHM@ZIF-8 increased with DHM feeding.

## Sample Availability

Samples of the ZIF-8 and DHM@ZIF-8 are available from the authors.
