# Supplementary material for: Pro-Apoptotic Effect of Zeolitic Imidazolate Framework-8 (ZIF-8)-Loaded Dihydromyricetin on HepG2 Cells
Source: Molecules. 2022 Aug 26;27(17):5484. doi: 10.3390/molecules27175484 (PMC9458003; doi:10.3390/molecules27175484)
Supplement: Supplementary file 1 [file molecules-27-05484-s001.zip › molecules-1850397-supplementary.pdf]

# Pro-Apoptotic Effect of Zeolitic Imidazolate Framework-8 (ZIF-8)-Loaded Dihydromyricetin on HepG2 Cells

**Xiao Mi [1,†], Juan Lu [1,†], Mingran Dong [1], Yang Lou [1], Xia Zhan [2,*] and Xi Chen [1,*]**

[1] Institute of Medicinal Plant Development, Chinese Academy of Medical Sciences, Peking Union Medical College, Beijing 100193, China
[2] Key Laboratory of Cleaner Production and Integrated Resource Utilization of China National Light Industry, Beijing Technology and Business University, Beijing 100048, China
* Correspondence: chenxi@implad.ac.cn (X.C.); zhanxia@th.btbu.edu.cn (X.Z.)
† These authors contributed equally to this work.

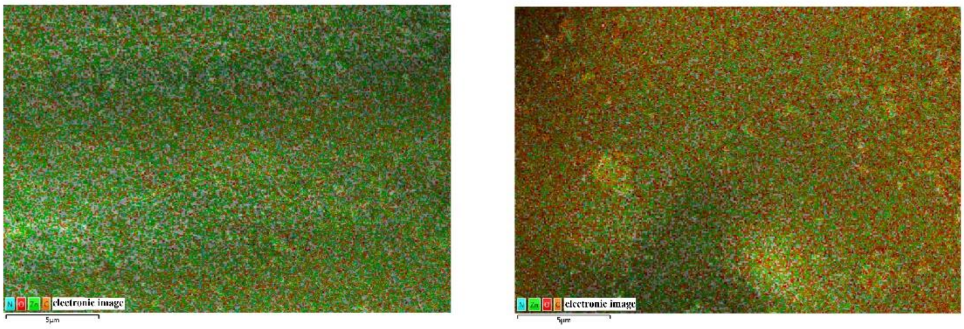

ZIF-8 DHM@ZIF-8

**Figure S1.** The elemental dispersive spectrum (EDS) of ZIF-8 and DHM@ZIF-8.

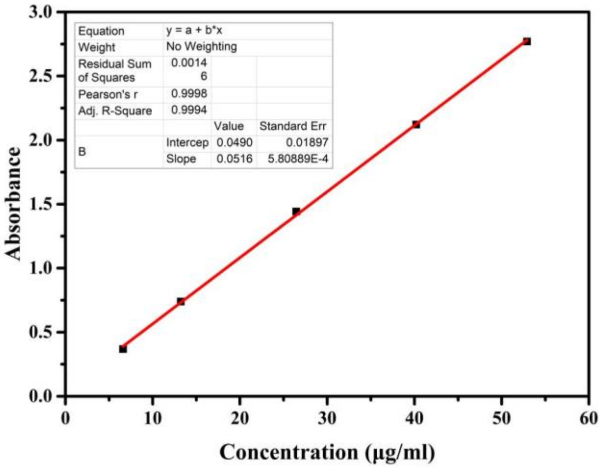


**Figure S2.** Standard curve of DHM solution detected by UV spectrophotometer at 290 nm.

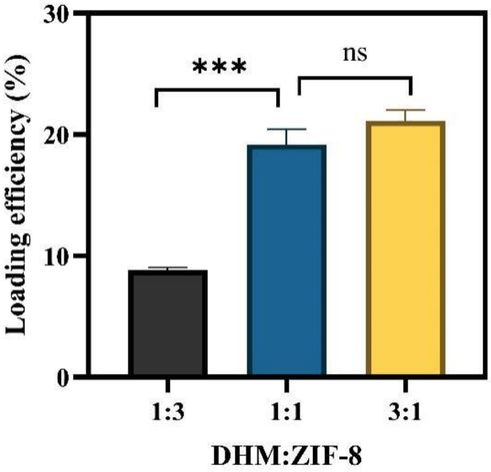


**Figure S3.** The loading efficiency of DHM@ZIF-8 increased with DHM feeding, *** $p < 0.001$.
